# Investigating the suitability of a transponder-based birth monitoring system attached to the vulva of a mare

**DOI:** 10.14202/vetworld.2023.2451-2456

**Published:** 2023-12-20

**Authors:** Hannah Lindinger, Axel Wehrend

**Affiliations:** Clinic for Obstetrics, Gynaecology and Andrology of Small and Large Animals, Justus-Liebig University, Giessen, Germany

**Keywords:** birth alarm, mare, parturition, transponder system

## Abstract

**Background and Aim:**

In horse breeding, birth monitoring is an important factor in minimizing losses during parturition. Although different birth monitoring systems are available for this purpose, the current literature lacks systematic suitability analyses. This study aimed to address this gap in the literature. In order to achieve this, we examined a large number of foaling mares to assess the suitability of a transponder-based birth monitoring system attached to the vulva.

**Materials and Methods:**

Seventy warmblood mares were observed during foaling, and 86 foals were born during the foaling seasons of 2021 and 2022. Video surveillance in the foaling stable provided video recordings of births. This allowed the opportunity to assess the birth monitoring system’s reporting accuracy. The exact times and reasons for each alarm were documented and the proportions of correctly detected births, false alarms, and unrecognized births were calculated.

**Results:**

Overall, 96.5% of foalings were correctly detected using the birth monitoring system, with a sensitivity rate of 96% and a specificity rate of 91%. False alarms were primarily caused when a mare rubbed her tail against the stable walls.

**Conclusion:**

These data suggest that the tested transponder is well suited for monitoring the birth of mares. However, it is recommended that this method should be used in combination with other birth monitoring methods because not all births were detected correctly.

## Introduction

The gestational length is widely variable in mares and may range from 320 to 360 days [1]. It is difficult to accurately predict the time of foaling on the sole basis of external physical changes. Compared with other domestic mammals, indicators such as relaxation of pelvic ligaments, mammary gland development, or increased vulva length may be only marginally visible and highly variable among mares, both in terms of temporal changes and individual manifestation [2–4]. Timely intervention in cases of dystocia, avoiding illnesses or losses of the mare and foal, and monitoring the timely intake of colostrum by the foal are all important reasons for prioritizing birth monitoring and reliably predicting the time of foaling [3, 5, 6].

Close visual monitoring of mares is very time-consuming and entails high personnel costs, as most mares give birth at night [7]. Therefore, continuous in-person birth monitoring is not feasible in most cases. Alternative methods for predicting the time of birth include measuring the body temperature of the mare [8, 9] and analyzing mammary secretions. However, mammary secretion analysis is more beneficial for predicting when a mare will not foal [10, 11], and both methods are more helpful in predicting the day of birth than the exact onset of birth. In order to address this problem, several birth monitoring systems have been designed to trigger a signal when the mare is in labor. However, little scientific research has been conducted on their suitability, practical applicability, and accuracy, all of which are clinically relevant for the practical implementation of birth monitoring.

In 1989, the first transponder-based birth monitoring system attached to a mare’s vulva was commercially available [12]. This system consists of a transmitter and a receiver. The transponder consists of a magnet attached to a slot in the device and a loop connected to the magnet. The transponder was then sutured onto the left labia, and the loop connected to the magnet was sutured onto the right labia. When the transponder device is first attached to the mare’s vulva, the magnet is already located in the slot on the transponder device. During parturition, the rima vulvae are mechanically spread by the passage of the fetal membranes, causing the magnet to separate from its base. This triggers a signal sent to the receiver, which triggers an alarm signal itself or through a connected (mobile) phone. The main advantage of this system is that there is no need for continuous monitoring of mares since an alarm is activated at the time of birth. To date, however, little research has investigated the effectiveness of this birth monitoring system in practice.

de Amorim *et al*. [11] tested the system in 37 standardbred mares. In 84% of births, an alarm corresponding with a foaling event was triggered by the system, while 16% of mares foaled without an alarm being initiated. In addition, the authors recorded four false alarms (i.e., alarms triggered without a mare being in foal) within the observation period. This was mainly caused by rubbing the tail and perineal area against the stall wall.

In addition to the lack of sensitivity data, a major shortcoming of the birth monitoring system is the fact that no alarm is triggered in the case of dystocia in which the fetus does not enter the birth canal (for example, when the fetus is transverse). This is considered to be a weakness of the system as it may have fatal consequences for the mare and unborn foal [3]. Therefore, the transponder system is often combined with other birth monitoring methods, such as camera monitoring or direct human observation [13].

The aim of this study was to test the transponder on a larger sample of foaling events to determine its suitability for birth monitoring in mares and to analyze the sensitivity and specificity of the system.

## Materials and Methods

### Ethical approval

This study was conducted in a veterinary practice for horses in Oldenburger Münsterland, Lower Saxony, Germany. All mares in the care of veterinary practice employees were treated according to the animal care guidelines of the Federation of Animal Science Societies [14]. These mares were fitted with a birth monitoring system and monitored at the request of their owners in the context of veterinary services. This is an evaluation of the parameters collected in the context of veterinary services. In accordance with the German Animal Welfare Act, it does not require ethical approval.

### Study period and location

This study was conducted during the 2021 and 2022 foaling seasons in a veterinary practice for horses in Oldenburger Münsterland, Lower Saxony, Germany.

### Animals

In the 2021 and 2022 foaling seasons, 86 births from 70 warmblood mares were examined. Of these mares, 16 were observed in both years, 15 were maiden mares, and 55 were pluriparous. The mares had a mean age of 11.49 ± 4.7 years (range, 4–22 years).

### Monitoring systems

Mares were individually stabled in 6 × 4 m or 4 3 m foaling stables, which were continuously monitored under video surveillance. The Sigloo foaling monitoring system [15], which functions in the same way as the Jan Wolters foaling system [12], was used to monitor birth. During parturition, the magnet is separated from the base of the transponder as the rima vulvae spread through the fetal membrane. As a result, a signal is sent to the receiver, which triggers an alarm and is transmitted to a mobile phone.

The exact date and time of insemination were recorded for each mare. The transponder was attached to the vulva of the mare approximately seven days before the estimated date of birth. This was based not only on a gestational length of 335 days, but also on external physical changes observed in mares nearing birth. Primarily, this referred to changes in the udder, as determined by the size and consistency of the udder, as well as filling of the teats and “waxing up” of the teat orifices. Physical changes, such as softening of pelvic ligaments and labia, were also evaluated. In view of the individual variation of these factors, the transponder was attached at least 7 days before the estimated date of parturition in mares, which did not show the abovementioned physical changes. When the device was fitted, the mares were restrained in an examination stand, where the tail was wrapped in a clean bandage and held by an assistant. The vulva was cleaned with water and dried with a paper towel. Subsequently, superficial disinfection with mucosa-compatible alcohol was performed, which was removed with a paper towel after a short exposure period. If the vulva was initially heavily soiled, the procedure described above was repeated.

**Figure-1:**
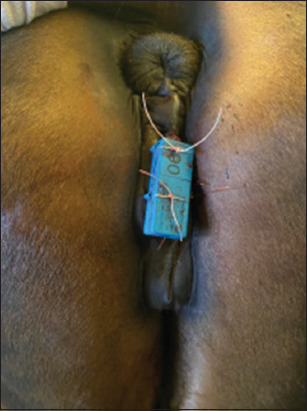
Transponder sutured to a pluriparous mare.

The exact position of the transmitter was determined individually for each mare depending on the size and anatomy of the vulva and vestibulovaginal canal (Figure-1). Once the correct position of the transmitter (approximately 1.5–2 cm from the rima vulvae) was established, 3–5 mL of a local anesthetic depot (Lidocainhydrochlorid 20 mg/mL injection solution for animals not intended for slaughter, Bela-Pharm GmbH and Co. KG, Vechta, Deutschland; Procainhydrochlorid 20 mg/mL injection solution for animals intended for slaughter, Richter Pharma AG, Wels, Österreich) was injected into the left labia. The anesthetic was administered at two locations (approximately 2 cm from each other) using a 23-G needle. Only one anesthetic depot was required on the right labium, and the needle was placed in a medial to lateral direction upon injection.

Subsequently, the left labium was punctured in the region of the local anesthetic depots using an 18-G needle moving from the medial to the lateral orientation. To fix the transponder device to the labia, a 6 metric suture was threaded through the eyelets provided on the transmitter and anchored with a surgical knot. Attachment of the loop connected to the magnet was executed according to the same principle on the right labium.

As soon as the birth detector was attached to the vulva of the mare, the general condition of the mares and the correct position of the transponder device were assessed twice daily. Rectal body temperature was recorded once daily as part of the routine examination. In addition, we assessed feed intake behavior and movement in the stable. Visual examination of the rima vulvae for signs of vaginal discharge was performed.

If an alarm was triggered by the birth monitoring system of one of the mares, two obstetricians, including at least one veterinarian, arrived at the foaling stable within minutes. The mare whose transmitter had triggered the alarm was located and examined to determine whether she was in labor or a false alarm had occurred. In addition, the foaling stable was video-monitored (1080P model from ieGeek, Hong Kong). Three cameras were installed in such a way that the entire stable could be observed. The video recordings were connected to the cameras, which allowed a retrospective assessment of the situation that triggered the alarm.

### Statistical analysis

We collected and analyzed data using Microsoft Office Excel 10 software (Microsoft Corp., Washington, USA). The time and reason for initiation were documented for each alarm. The proportion of correctly detected and reported births, false alarms, and unrecognized births were calculated as a percentage. All alarms triggered without a mare in labor were considered false alarms. Foaling events that did not trigger an alarm from the transmitter were also recorded as false alarms, indicating that the transponder device failed to recognize and report the birth. The sensitivity and specificity of the birth monitoring system were calculated as follows:

Sensitivity = births correctly detected by the transponder/(births correctly detected by the transponder + births not detected by the transponder).

Specificity = births correctly detected by the transponder/(births correctly detected by the transponder + false alarms [without a mare at parturition]).

## Results

During the 2021 foaling season, the transponder was not able to detect one birth (Table-1). In the present case, the mare suffered a complete perineal laceration during parturition in the 2018 foaling season. The perineal laceration was surgically treated in 2019, and the mare was successfully inseminated in 2020. The perineum reconstruction resulted in the formation of a pocket at the dorsal commissure of the vulva. It was not possible to open this mucosal fold wide enough before parturition without risking injury to the rectum, of which the caudoventral end extended far ventrally into the vestibulovaginal canal due to the reconstruction. The front hooves of the foal became stuck in this mucosal fold during the expulsion phase, which prevented the transponder device from initiating an alarm.

**Table-1:** Overview of parturition in mares detected and not detected by the birth monitoring system in relation to the total number of monitored births in the 2021 and 2022 foaling seasons.

Foaling season	Correct triggering of the alarm, corresponding with a foaling event	False alarm	Total births

		Failure to detect a birth without triggering an alarm	
		
	n (%)	n (%)	n (%)
Foaling season 2021	39 (97.5)	1 (2.5)	40 (100)
Foaling season 2022	44 (95.6)	2 (4.3)	46 (100)
Total	83 (96.5)	3 (3.4)	86 (100)

During the 2022 foaling season, the transponder did not correctly detect two parturitions (Table-1). One case was the stillbirth of an externally completely developed foal on the 336^th^ day after the last insemination. The cause of antepartum fetal death was likely to be umbilical cord torsion. During a routine examination, small amounts of amniotic fluid were observed to be discharged from the mare’s vulva, but no fetal membranes or parts of the fetus were visible in the rima vulva. Subsequent examinations showed that the mare was in labor, but the fetus was no longer alive. The deceased foal was delivered by manual obstetrics.

Dystocia was a factor in the case of the second unrecognized birth. On the 349^th^ day of gestation, the mare was found in acute shock in the stable. There were no externally noticeable physical signs that the mare was in labor. A vaginal examination revealed that the foal presented in the transverse position, lower stance, and flexed head posture with bilateral flexion of the carpal joint. Due to the poor prognosis of the mare, a fully developed foal was delivered by sectio brutalis.

In total, eight alarms were activated, during which it was determined that the mare in question was not in labor (Table-2). In all eight instances, this was caused by the mare rubbing the perineal area and tail against the stable wall, which released the magnet from its slot in the transmitter, thus triggering the alarm. In all of these cases, it was possible to reinsert the magnet into the transmitter base, making the transponder instantly operational again.

**Table-2:** Overview of parturition in mares detected by the birth monitoring system and the triggering of an alarm without the mare being in labor, in relation to the total alarms triggered by the birth monitoring system in the 2021 and 2022 foaling seasons.

Foaling season	Correct triggering of the alarm, corresponding with a foaling event	False alarm	Total alarms

		Triggering an alarm without a foaling event	
		
	n (%)	n (%)	n (%)
Foaling season 2021	39 (88.6)	5 (11.4)	44 (100)
Foaling season 2022	44 (93.6)	3 (6.4)	47 (100)
Total	83 (91.2)	8 (8.8)	91 (100)

Two of the false alarms in the 2021 foaling season and one in 2022 (Table-2) were triggered by different mares shortly after the transponder device had been sutured in place. One of these mares foaled one night after the false alarm was triggered; the other two after 10 and 14 days, respectively.

One false alarm in the 2021 foaling season and another false alarm in the 2022 foaling season (Table-2) were triggered by mares in the early hours of the morning without any apparent reason. One of these mares foaled 6 days after the false alarm was triggered, and the other mare foaled 3 days after the false alarm.

The two remaining false alarms recorded during the 2021 foaling season (Table-2) were activated one night by a single mare, approximately 2 h apart. The mare showed significant colic symptoms, including restlessness, sweating, rolling, and inappetence. In addition, the examination found that the mare was not in labor. Four days later, a healthy foal was delivered without any complications.

The last false alarm during the 2022 foaling season (Table-2) was triggered by a mare for which another alarm was recorded 2 h later. During the second alarm, the mare was in the expulsion phase of parturition, indicating an actual birthing event. For all mares that experienced a false alarm during the 2021 and 2022 foaling seasons, a correct alarm was indeed activated at the moment of actual parturition. A sensitivity of 96% and a specificity of 91% were calculated for the birth detector.

## Discussion

The rates of dystocia in mares range from 2% to 13% [16]. During severe complications of parturition, human intervention is necessary to ensure the survival of the foal and the mare. Dystocia is regarded as an emergency situation where prompt identification and application of appropriate obstetric interventions is essential. Therefore, it is crucial to predict the time of birth precisely and to monitor the mare during parturition. In addition to ethical considerations and the emotional value of mares and foals for the horse owner, the economic aspect of horse breeding should also be considered [3, 17]. In case of complications, breeders may face significant financial losses. Although various birth monitoring systems have been commercially available for decades, their efficacy has been largely neglected in scientific literature. To the best of our knowledge, this is the first study that examined transponders attached to a large group of mares.

To date, only one comparable study has investigated the same system in a smaller sample of animals. de Amorim *et al*. [11] were able to register an alarm associated with parturition in only 31 of 37 mares (84%). The authors attributed this to a weak receiver signal, detachment of the suture from the magnet, or suture unraveling due to rubbing of the perineal area. This result is significantly lower than the 96.5% success rate observed in this study. In addition, the reasons for non-detected births varied between the two studies. While de Amorim *et al*. [11] associated non-detected foaling events with technical problems or incorrect attachment of the device, this was not observed in the present study. Technical failure was not found to cause any false alarms, and none of the undetected parturitions were associated with eutocia.

This reinforces the hypothesis that the system cannot guarantee reliable alarm triggering in cases of dystocia; therefore, it is not feasible to rely solely on the birth monitoring system for parturition detection in all cases [13]. In the present study, births for which no alarm was triggered were detected by additional camera surveillance or by direct visual observation of the mare. This further supports the argument that relying solely on a transponder is insufficient for detecting all births [13]. Future studies should focus on whether a combination of different birth monitoring systems improves the safety of the prediction or detection of birth in mares.

Eight false alarms (8.8% of all false alarms) were recorded in the present study compared with 11% observed in the work of de Amorim *et al*. [11]. The causes of the erroneously activated alarms were similar in both studies; false alarms were often preceded by instances in which the mares rubbed the perineal area against the stable wall, resulting in the magnet detached from the slot in the transmitter base. This may suggest that some mares experienced the birth detector as irritating and attempted to rub it off. This may be the case for the false alarms that occurred shortly after the birth detector was sutured in place, as well as for those that were triggered without an apparent cause.

One false alarm detected in this study was triggered by a mare 2 h prior to parturition. In the present case, rubbing of the perineal area against the stable wall during the opening phase of parturition, as described by various authors [9, 18], may explain the occurrence of this false alarm.

To prevent any adverse impact on the mares due to the birth monitoring system, Müller *et al*. [17] emphasize the need for the system to be non-invasive. The transponder studied does not fulfill this requirement. Due to the requirement to suture the transmitter to the mare’s vulva, the birth monitoring system is relatively invasive. Nevertheless, in the present study, it was possible to attach the device to the vulva in all mares. However, for nonspecific reasons, de Amorim *et al*. [11] chose not to utilize the system in three mares. According to the authors, the act of suturing represents a vulnerability of the system, as the sutures may unravel from the labia, leading to a situation where the system may not consistently trigger. This could not be confirmed in the present study, but the transmitters were examined once daily for correct position and the fixation was corrected if necessary. Whether (and how often) examinations of the correct position and fixation of the transmitters were performed by de Amorim *et al*. [11] was not described.

In evaluating the reviewed birth monitoring system, it is also necessary to consider the timing at which an alarm is initiated. The system triggers an alarm only during the expulsion phase [17], i.e., when the mare has progressed significantly in the birthing process. Consequently, it is imperative that the obstetricians are in close proximity to the stable, given the mare’s relatively brief expulsion phase [19], to use this system appropriately. This constraint limits the widespread use of this birth monitoring system. Not all mares are stabled in such a way that an obstetrician can be on site within a few minutes. In such cases, it may be more appropriate to use a system capable of predicting parturition at an earlier time.

Given its sensitivity and specificity of 96% and 91%, the transponder can be described as a good monitoring system for parturition in mare. To date, similar characteristic values have only been documented for body temperature measurements in the context of birth monitoring. It should be noted, however, that the purpose of recording body temperature in a mare is not to detect a mare in the expulsion phase of parturition, but to predict whether parturition will occur within a defined period of time.

Korosue *et al*. [8] reported a specificity of 77% for days 1–5 before parturition and a sensitivity of 71% on the evening before parturition for internal body temperature measured using a microchip implanted in the neck. Auclair-Ronzaud *et al*. [9], using a microchip implanted in the neck, achieved a sensitivity of 96.9% and a specificity of 95% when comparing the average temperature of the 12 h before parturition to the temperatures from the preceding days at the same time of day.

## Conclusion

This study demonstrates that the transponder device under investigation is well suited for monitoring birth in mares. This study is the first to compute the sensitivity and specificity of a birth monitoring system using a large sample of animals. However, since the system did not detect all births, it is recommended to be used in conjunction with another method for monitoring parturition in mare.

This study primarily focused on the evaluation of the birth monitoring system. The safety and reliability with which the transponder detected and reported a birth were tested. In future research, detecting behaviors in the peripartum period that can enhance the reliability of detection and/or prediction of the time of birth would be scientifically valuable.

## Authors’ Contributions

HL: Collected the data, performed the analysis, and drafted the manuscript. AW: Conceived and designed the study and drafted and revised the manuscript. Both authors have read, reviewed, and approved the final manuscript.
